# AI Advancements in Healthcare: Medical Imaging and Sensing Technologies

**DOI:** 10.3390/bioengineering12101026

**Published:** 2025-09-26

**Authors:** Mohammed A. Al-masni, Kanghyun Ryu

**Affiliations:** 1 Department of Artificial Intelligence and Data Science, College of Artificial Intelligence Convergence, Sejong University, Seoul 05006, Republic of Korea; 2 Intelligence and Interaction Research Center, Korea Institute of Science and Technology (KIST), Seoul 02792, Republic of Korea; 3 AI-Robotics, KIST School, University of Science and Technology, Seoul 01811, Republic of Korea

## 1. Introduction

Artificial intelligence (AI), broadly defined as algorithms capable of self-learning patterns from large-scale data, has emerged as one of the most transformative technologies in modern healthcare [1]. Advances in medical imaging and sensing have created unprecedented opportunities for data-driven analysis. Imaging modalities such as magnetic resonance imaging (MRI), computed tomography (CT), and X-ray offer detailed visualization of structural changes in the body, while sensing techniques such as electroencephalography (EEG), electrocardiography (ECG), and electromyography (EMG) provide complementary measurements of physiological activity. Together, these modalities generate complex datasets ideally suited for AI-driven modeling. When integrated with advanced computational methods, they extend far beyond traditional diagnostic roles to enable earlier disease detection, personalized treatment planning, and continuous monitoring of health trajectories.

Looking forward, the role of AI in healthcare will hinge on developing systems that are not only innovative but also trustworthy. The FUTURE-AI framework [2] highlights six guiding principles—fairness, universality, traceability, usability, robustness, and explainability—as essential for ensuring safe and reliable clinical adoption.

This Special Issue of *Bioengineering*, titled “AI Advancements in Healthcare: Medical Imaging and Sensing Technologies”, brings together five innovative contributions [3,4,5,6,7] that collectively represent the state of the art in this rapidly evolving field. The selected works span a broad spectrum of imaging modalities—from video silhouettes and dermoscopy to MRI, chest X-ray, and intraoral photography—and apply a diverse range of AI methodologies, including novel convolutional neural network (CNN) architectures, quantum-enhanced classifiers, interpretable brain-aging biomarkers, vision–language pipelines, and foundation segmentation models. Each article situates its technical advances within a clinically meaningful application, addressing challenges such as neurological gait assessment, dermatological cancer screening, neurodegenerative disease staging, radiological reporting, and restorative dentistry. However, it should be noted that although the theme of this Special Issue encompasses both imaging and sensing technologies, the final collection of accepted articles is focused exclusively on imaging applications. No contributions addressing sensing modalities were included among the published works, and we hope that future editions of this Special Issue will also attract submissions that highlight AI-driven advances in sensing technologies.

Collectively, these studies highlight not only the innovative use of AI technologies but also their translational potential, showing the alignment of computational advances with pressing clinical needs. Table 1 provides a comparative overview of the five articles, summarizing their clinical aims, imaging modalities and datasets, methodological innovations, validation strategies, and interpretability features.

## 2. Overview of Published Articles

This section provides an overview of the five contributions included in this Special Issue. Each subsection briefly summarizes the motivation of the work, the technical AI advancements presented, and the clinical implications, including considerations of feasibility and interpretability.

### 2.1. Functional Assessment Using Gait Dynamics

The first contribution in this Special Issue focuses on gait analysis [3] as a proxy for functional assessment in neurological disorders. The study introduces a novel method that transforms binary silhouette sequences of walking subjects into sinograms by projecting pixel intensities over a range of angular directions. This process yields motion-encoded maps in which temporal dynamics of gait are compactly represented as continuous angular patterns. These sinogram representations are subsequently processed by a 1D-CNN equipped with an assisted knowledge learning strategy, enabling the model to capture both local temporal fluctuations and global gait signatures. Evaluated on the INIT GAIT dataset [8,9], the framework demonstrated consistently high accuracy across different classification schemes, including both frame-level analysis and subject-level aggregation through majority voting. A key advantage of the method is that the sinogram representation makes the underlying gait dynamics easier to interpret, as it directly encodes angular motion in a form that clinicians can relate to observable movement patterns. By combining this representation with low-cost video input, the study demonstrates a practical pathway toward the development of scalable tools that could support neurological screening and rehabilitation in real-world settings.

### 2.2. Dermatological Image Analysis

The second contribution addresses the growing demand for automated computer-aided diagnosis (CAD) in dermatology, focusing on multi-class classification of skin lesions [4]. To enhance generalizability, the study draws on three widely used public datasets—PAD-UFES-20-Modified [10,11], ISIC-2018 [12], and ISIC-2019 [13]—thereby capturing a broad spectrum of lesion appearances and acquisition conditions. The proposed framework integrates a CNN with an autoencoder to extract both local image features and compact latent representations. These features are then processed by a quantum support vector machine (QSVM), which is designed to exploit high-dimensional feature spaces for complex decision boundaries.

A critical aspect of the work lies in its comprehensive preprocessing strategy, including image normalization and enhancement, which ensures consistent input quality across heterogeneous datasets. By combining classical deep learning with quantum-inspired classification, the study illustrates a promising direction for dermatological CAD systems, particularly in settings where robustness across multiple datasets is essential. Although the use of QSVM introduces challenges for interpretability, this limitation opens avenues for future research on integrating explainable AI tools to increase clinical trust.

### 2.3. AI Biomarkers for Neurodegenerative Diseases

The third contribution presents an innovative approach to quantifying brain aging at a regional level, with direct implications for neurodegenerative disease research and early diagnosis [5]. Building on the concept of brain-age prediction models, the authors developed the Regional Brain Aging Disparity Index (RBADI), which captures localized deviations between predicted and chronological age across distinct brain regions. The method relies on a deep learning-based brain-age model trained on a large-scale cohort from the UK Biobank [14], encompassing over sixteen thousand healthy individuals. To improve interpretability, the study employs a multi-stage Shapley value approximation, enabling the attribution of brain-age predictions to anatomically meaningful regions and therefore grounding the RBADI in established neuroanatomy.

The framework was externally validated on independent datasets from the Alzheimer’s Disease Neuroimaging Initiative (ADNI) [15] and the Parkinson’s Progression Markers Initiative (PPMI) [16], demonstrating its applicability across multiple neurological conditions. Beyond diagnostic staging, RBADI was further linked to lifestyle and demographic factors, highlighting its potential as a population-level biomarker for understanding risk profiles. By integrating large-cohort training, external validation, and explainable AI techniques, this work illustrates how region-specific biomarkers can bridge the gap between computational models and clinically relevant insights into neurodegenerative disease progression.

### 2.4. Vision–Language Models for Radiological Reporting

The fourth contribution advances the integration of AI into radiological workflows by tackling the complex task of automated report generation from chest X-rays [6]. Unlike conventional pipelines that focus narrowly on classification or segmentation, this study introduces the Integrated Hierarchical Radiology Assistant System (IHRAS), an end-to-end framework designed to produce clinically meaningful textual reports. The system combines multiple AI components: a CNN for multi-label disease classification, Grad-CAM for visual explanation, SAR-Net for anatomical region segmentation, and a large language model (LLM) guided by structured prompt engineering. Importantly, the language component is aligned with established ontologies such as SNOMED CT, ensuring consistency with medical standards.

The framework is built on the large-scale NIH ChestX-ray dataset [17] and emphasizes not only diagnostic performance but also the coherence, relevance, and faithfulness of the generated reports. By fusing image classification, region-level anatomical context, and language modeling, IHRAS demonstrates how vision–language integration can move AI systems closer to supporting radiologists in daily practice. The inclusion of interpretability mechanisms, both visual (Grad-CAM) and linguistic (standardized terminology), strengthens transparency and clinical trust, positioning this work as a significant step toward practical AI adoption in radiology reporting.

### 2.5. Foundation Models for Dental Imaging

The final contribution in this Special Issue explores the application of foundation models to restorative dentistry, with a focus on automating tooth and shade-guide segmentation from intraoral photographs [7]. Accurate shade matching is a critical step in dental restoration, yet it remains prone to variability due to lighting conditions, subjective assessment, and manual delineation. To address this challenge, the study evaluated multiple variants of the Segment Anything Model 2 (SAM2), including tiny, small, base+, and large configurations against a conventional U-Net baseline. By fine-tuning SAM2 on a curated dataset of intraoral images, the authors demonstrated the capacity of large-scale pretrained segmentation models to adapt effectively to highly specialized clinical tasks.

A noteworthy aspect of the study is its emphasis on not only boundary precision but also color fidelity, assessed through perceptually calibrated metrics such as CIELAB and ΔE00. This dual evaluation shows the practical importance of segmentation quality for ensuring accurate shade selection in restorative workflows. While the dataset was limited to a single-center collection, the findings highlight the translational promise of adapting foundation models to dentistry, a domain where annotated data are typically scarce. By leveraging pretrained general-purpose vision models, this work points toward the more efficient development of AI tools for specialized yet clinically significant applications.

## 3. Conclusions

This Special Issue of *Bioengineering* shows the breadth and depth of current advances in artificial intelligence for healthcare, encompassing functional assessment, dermatological diagnosis, neuroimaging biomarkers, radiological reporting, and dental applications. Across these diverse domains, the featured studies demonstrate how methodological innovations, ranging from novel signal representations and hybrid learning pipelines to interpretable biomarkers, vision–language integration, and the adaptation of foundation models, can be translated into clinically meaningful outcomes. These contributions highlight that progress in healthcare AI must extend beyond improvements in accuracy to also embrace interpretability, standardization, and practical usability, ensuring that technological developments are aligned with the realities of clinical practice.

## Figures and Tables

**Table 1:** A summary of the five articles published in this Special Issue, titled “AI Advancements in Healthcare: Medical Imaging and Sensing Technologies”.

Ref.	Clinical Aim	Modality/Dataset	Methodology	Validation Setup	Interpretability
Al-masni et al. [3]	Screening and assessment of gait abnormalities	2D video silhouettes/INIT GAIT [8,9]	Novel silhouette sinogram representation and 1D-CNN with assisted knowledge learning	Two schemes: frame-level classification and subject-level majority voting	Intrinsic interpretability through sinogram encoding of angular motion
Khan et al. [4]	Automated CAD for melanoma and other skin lesion types	Dermoscopy/PAD-UFES-20-Modified [10,11], ISIC-2018 [12], ISIC-2019 [13]	Hybrid CNN–autoencoder with advanced preprocessing; quantum SVM as final classifier	Cross-dataset benchmarking; class imbalance addressed via augmentation	Limited intrinsic interpretability; potential for XAI to enhance clinical trust
Wu et al. [5]	Region-level aging analysis and staging of neurodegeneration	T1-weighted MRI/UK Biobank [14], ADNI [15], PPMI [16]	DL-based brain-age prediction with multi-stage Shapley value approximation → RBADI biomarker	Train/val/test split on UKB; external validation on ADNI and PPMI	Strong interpretability via regional Shapley attribution; anatomically grounded RBADI
Rodrigues et al. [6]	AI pipeline for diagnostic reporting	Chest X-ray/NIH CXR [17]	CNN multi-label classifier (14 diseases) + Grad-CAM + SAR-Net anatomical segmentation + LLM (DeepSeek-R1, CRISPE prompts, SNOMED CT)	Internal benchmarking across diverse demographic subgroups	Multi-level interpretability: visual explanations (Grad-CAM), anatomical context (SAR-Net), and standardized terminology (SNOMED CT)
Han et al. [7]	Tooth and shade-guide segmentation for shade matching	Intraoral photographs/private dataset	Fine-tuned Segment Anything Model 2 (tiny/small/base+/large) vs. UNet baseline	Held-out test split; comparative evaluation across SAM2 scales	Robust interpretability via boundary precision and quantitative color fidelity (CIELAB, ΔE00)

## Abbreviations

The following abbreviations are used in this manuscript:

AI	Artificial Intelligence
ADNI	Alzheimer’s Disease Neuroimaging Initiative
CAD	Computer-Aided Diagnosis
CNN	Convolutional Neural Network
CT	Computed Tomography
CXR	Chest X-ray
ECG	Electrocardiography
EEG	Electroencephalography
EMG	Electromyography
Grad-CAM	Gradient-Weighted Class Activation Map
IHRAS	Integrated Hierarchical Radiology Assistant System
ISIC	International Skin Imaging Collaboration
LLM	Large Language Model
MRI	Magnetic Resonance Imaging
PPMI	Parkinson’s Progression Markers Initiative
QSVM	Quantum Support Vector Machine
RBADI	Regional Brain Aging Deviation Index
SAM	Segment Anything Model
SAR-Net	Structure-Aware Relation Network
SVM	Support Vector Machine
XAI	Explainable Artificial Intelligence
